# Supplementary material for: Content-rich biological network constructed by mining PubMed abstracts
Source: BMC Bioinformatics. 2004 Oct 8;5:147. doi: 10.1186/1471-2105-5-147 (PMC528731; doi:10.1186/1471-2105-5-147)
Supplement: Additional File 2 — The original results of the above study (non-essential files are deleted to keep the file size under the limit set by BMC bioinformatics). [file 1471-2105-5-147-S2.bz2 › chilibotAdditionalFile2/dip05/85ID8702708E312/html/TRAF1_TRAF2.html]

 


 **TRAF1** and **TRAF2** 
  
Found 193 abstracts in PubMed, retrieved 05.  
 

 What does Google say? 
 PDF only 
| .edu only 

---

**Interactive relationship** (e.g. stimulation, inhibition, etc)

**Non-interactive relationship** (e.g. studied together, co-existance, homology, etc.)

- We have previously reported that the HCV NS5A protein interacts with TRAF  [ **TRAF1** ]  domain of TNF receptor associated factor 2  [ **TRAF2** ]   **TRAF2** .  Ref: 12796506 J Biol Chem< MedlineTA>J Biol Chem,
